# Distinct Functional Connectivities Predict Clinical Response with Emotion Regulation Therapy

**DOI:** 10.3389/fnhum.2017.00086

**Published:** 2017-03-03

**Authors:** David M. Fresco, Amy K. Roy, Samantha Adelsberg, Saren Seeley, Emmanuel García-Lesy, Conor Liston, Douglas S. Mennin

**Affiliations:** ^1^Department of Psychological Sciences, Kent State UniversityKent, OH, USA; ^2^Department of Psychiatry, Case Western Reserve University School of MedicineCleveland, OH, USA; ^3^Department of Psychology, Fordham UniversityBronx, NY, USA; ^4^Department of Psychology, University of ArizonaTucson, AZ, USA; ^5^The Graduate Center, City University of New YorkNew York, NY, USA; ^6^Hunter College, City University of New YorkNew York, NY, USA; ^7^Department of Psychiatry, Weill Cornell Medical CollegeNew York, NY, USA

**Keywords:** generalized anxiety disorder, major depressive disorder, worry, somatic anxiety, decentering, resting state functional connectivity

## Abstract

Despite the success of available medical and psychosocial treatments, a sizable subgroup of individuals with commonly co-occurring disorders, generalized anxiety disorder (GAD) and major depressive disorder (MDD), fail to make sufficient treatment gains thereby prolonging their deficits in life functioning and satisfaction. Clinically, these patients often display temperamental features reflecting heightened sensitivity to underlying motivational systems related to threat/safety and reward/loss (e.g., somatic anxiety) as well as inordinate negative self-referential processing (e.g., worry, rumination). This profile may reflect disruption in two important neural networks associated with emotional/motivational salience (e.g., salience network) and self-referentiality (e.g., default network, DN). Emotion Regulation Therapy (ERT) was developed to target this hypothesized profile and its neurobehavioral markers. In the present study, 22 GAD patients (with and without MDD) completed resting state MRI scans before receiving 16 sessions of ERT. To test study these hypotheses, we examined the associations between baseline patterns of intrinsic functional connectivity (iFC) of the insula and of hubs within the DN (anterior and dorsal medial prefrontal cortex [MPFC] and posterior cingulate cortex [PCC]) and treatment-related changes in worry, somatic anxiety symptoms and decentering. Results suggest that greater treatment linked reductions in worry were associated with iFC clusters in both the insular and parietal cortices. Greater treatment linked gains in decentering, a metacognitive process that involves the capacity to observe items that arise in the mind with healthy psychological distance that is targeted by ERT, was associated with iFC clusters in the anterior and posterior DN. The current study adds to the growing body of research implicating disruptions in the default and salience networks as promising targets of treatment for GAD with and without co-occurring MDD.

## Distinct Functional Connectivities Predict Clinical Response with Emotion Regulation Therapy

Generalized anxiety disorder (GAD) and major depressive disorder (MDD) are prevalent and impairing conditions when they occur alone. However, when they co-occur, GAD and MDD are associated with increased public health burden and are more treatment refractory (e.g., Whisman et al., 2000; Stein and Heimberg, 2004; Farabaugh et al., 2010; Newman et al., 2010; Tully et al., 2013). Psychological models characterize GAD and MDD as conditions illustrative of a profound disruption between mind and body motivated by a fraught attempt to avoid unpredictability and acute intense emotionality brought about by actual or perceived threat (GAD) or actual or perceived loss/reinforcement deprivation (MDD). Specifically, individuals with GAD and/or MDD characteristically respond to aversive and often conflicting emotional and somatic experiences with the use of repetitive or perseverative reactive cognitive processes such as worry and rumination (Mennin and Fresco, 2013). These processes are enacted to create control and predictability, but instead more often result in vacillation between a worried or ruminative mind and chronically distressed body. When individuals are momentarily successful in staving off the aversive experience of strong emotional responses by invoking such self-evaluative processes, reliance on these self-evaluative processes is reinforced (Borkovec et al., 2004; Nolen-Hoeksema et al., 2008; Watkins, 2008; Newman and Llera, 2011; Mennin and Fresco, 2013, 2014; Olatunji et al., 2013). Similarly, behavioral activation models of depression posit that depressive rumination serves an avoidance function by promoting unhelpful self-reflection on one’s negative events instead of engaging behavioral actions to respond or resolve the particular circumstances (e.g., Ferster, 1973; Jacobson et al., 2001; Papageorgiou and Wells, 2004). As a result, worry and rumination are associated with considerable deficits in cognitive and behavioral responding (e.g., Lissek, 2012; Whitmer and Gotlib, 2012) as well as an inferior treatment response and greater relapse (e.g., Jones et al., 2008).

Although worry may temporarily reduce acute and intense somatic arousal, over the long run, it may actually promote persistent negative emotions and physical stress (e.g., Brosschot et al., 2006; Newman and Llera, 2011). For instance, ambulatory monitoring studies indicate that individuals with GAD as compared to healthy individuals have relatively higher heart rate (e.g., Hoehn-Saric et al., 2004). Similarly, laboratory studies utilizing worry induction methodology reveal that worry is associated with greater heart rate and skin conductance (e.g., Vrana and Lang, 1990; Lyonfields et al., 1995; Thayer et al., 1996). Relatedly, individuals with GAD evidence chronically low vagal tone especially in relation to worry (Thayer et al., 1996; Brosschot and Thayer, 2003; Hoehn-Saric et al., 2004; Brosschot, 2010), which may reflect a diminished physiological flexibility brought about by the physiologic changes induced by chronic anxiety. As a result of this diminished capacity for physiological self-regulation, individuals may become increasingly reliant on maladaptive strategies such as repetitive thought to manage distress (e.g., Hoehn-Saric et al., 2004). Similarly, the relationship between worry and physiological arousal may reflect the very way that individuals with GAD learn to cope with negative emotionality. Individuals with GAD commonly exhibit paradoxical acute anxiety when instructed to relax (e.g., Heide and Borkovec, 1984) and express greater success in coping with fear and sadness inducing probes with worry as compared to relaxation or neutral instructions (e.g., Llera and Newman, 2010).

In an effort to synthesize these transdiagnostic features of GAD and MDD, Mennin and Fresco (2013, 2014) have posited an emotion dysregulation model, in which these conditions are marked by heightened emotional experience (i.e., motivational intensity) coupled with repetitive and perseverative forms of self-referential thinking (i.e., worry, rumination, self-criticism) that serve as compensatory strategies to reactively escape or avoid strongly felt emotional and somatic experiences. The combination of these clinical features may reflect an underlying profile or endophenotype common to GAD and MDD, and may also account for the relative underperformance of otherwise efficacious treatments in resolving these conditions, particularly when comorbid (e.g., Olatunji et al., 2010). A greater focus on the underlying features of emotionality and self-referentiality may provide a more refined target of investigation than simply examining diagnostic symptoms of GAD. This model is congruent with transdiagnostic approaches to these conditions (e.g., Nolen-Hoeksema and Watkins, 2011) and is consistent with the Research Domain Criteria project (e.g., Insel et al., 2010), which represents an effort to leverage our knowledge of both normative and disordered human functioning at many levels of analysis (e.g., genetic, molecularly, neural, behavioral, socio-cultural, etc.,). Using an experimental therapeutics framework, the Research Domain Criteria promotes investigations of novel intervention principles for their action on biobehavioral targets in hopes of producing superior and enduring clinical improvement (Kozak and Cuthbert, 2016).

Negative self-referentiality and intense emotionality, the primary characteristics of the endophenotype posited in Mennin and Fresco (2013, 2014) emotion dysregulation model, correspond to two well researched neural networks: the *default network* (DN; e.g., Raichle et al., 2001) and the *salience network* (SN; e.g., Craig, 2009; Menon, 2015), respectively. In particular, self-referentiality is commonly associated with neural activation in the DN, regarded as a network associated with autobiographical, self-monitoring and social cognitive functions. The DN is anchored by activity in the medial prefrontal cortex (MPFC; narrative and autobiographical self) and the posterior cingulate cortex (PCC; experiential self-reflection; e.g., Buckner et al., 2008; Qin and Northoff, 2011; Brewer et al., 2013).

The MPFC is also implicated in the detection of emotionally salient stimuli (Morris et al., 1998; Phillips et al., 2003), agentic or aspirational self-reflection (Johnson et al., 2006, 2009), and determining whether beliefs are “acceptable” or “unacceptable” (Paulus and Stein, 2010). Similarly, the PCC has been implicated in self-reflection especially in relation to duties and obligations (Johnson et al., 2006, 2009). Psychiatric disorders are often marked by excessive activation of the DN thereby preventing or delaying purposeful activation of neural regions associated with executive control (e.g., Whitfield-Gabrieli and Ford, 2012) as well as undermining cognitive load and emotion regulation capacities (e.g., Brewer et al., 2011; Whitfield-Gabrieli and Ford, 2012). With respect to GAD and MDD, resting state fMRI studies clearly implicate disruption in DN regions (e.g., Hamilton et al., 2011, 2013; Chen and Etkin, 2013; Andreescu et al., 2014; Wang et al., 2016). Similarly, task-based studies examining trait levels of worry or depressive rumination (e.g., Paulus and Stein, 2010; Hamilton et al., 2011) or instructions to worry or ruminate (e.g., Cooney et al., 2010; Paulus and Stein, 2010; Ottaviani et al., 2016) demonstrate neural activations in nodes of the DN.

The SN governs our attention to the external and internal world (Menon and Uddin, 2010), and as such, integrates sensory, emotional and cognitive information to facilitate optimal communication, social behavior and self-awareness (Menon, 2015). A critical node of the SN is the insular cortex, which has been implicated in interoceptive awareness (e.g., Mussgay et al., 1999; Critchley et al., 2004; Craig, 2011; Farb et al., 2015), and afferent information that arises from anywhere and everywhere within the body (Cameron, 2001). Greater interoceptive awareness is associated with increased anxiety and panic, especially when those bodily sensations are catastrophized (Schandry, 1981; Ehlers and Breuer, 1992; Barlow, 2002; Pollatos et al., 2009; Paulus and Stein, 2010) during self-referential processing, such as in worry (e.g., Pollatos et al., 2009; Paulus and Stein, 2010). This self-referencing may thus exaggerate arousal (i.e., positive or negative). According to Paulus and Stein (2010), individuals with anxiety and depression exhibit a reduced signal to noise ratio of interoceptive afferents: interoceptive signals have a propensity to be interpreted negatively, resulting in increased sympathetic arousal, and in turn, increased escape or avoidance behaviors. Consequently, low fidelity interoceptive afferents result in overactive top-down brain modulatory areas (e.g., MPFC) that engage constantly to differentially amplify or attenuate body signals.

The insula is thought to also serve a role in evaluating the impact of stimuli on the body (Paulus and Stein, 2006), including generation and regulation of affective responses and detection of emotionally salient stimuli (Paulus and Stein, 2010). Focus has been on the right anterior insula (e.g., Critchley et al., 2004) but increasingly, evidence also indicates a role for the posterior insula (e.g., Simmons et al., 2013; Kuehn et al., 2016) as well as bilateral insulae (e.g., Stein et al., 2007; Avery et al., 2014). Neuroimaging studies support the contention that disorders such as GAD and MDD are associated with SN abnormalities. For instance, compared to healthy individuals, depressed patients show reduced connectivity between anterior insula and other nodes of the SN (Yuen et al., 2014). Consistent with the predictions of Paulus and Stein (2010), task-based studies with MDD and GAD patients consistently show hyperactivity of the anterior insula often accompanied by increased connectivity with nodes of DN including the PCC (e.g., Paulus and Stein, 2010; Hamilton et al., 2013; Yuen et al., 2014). Similarly, a recent study by Kaiser et al. (2015) found that in comparison to healthy control participants, patients with MDD evidenced increased connectivity of the MPFC to the insula and the strength of this connectivity was predictive of depression severity.

In contrast to the maladaptive relationship between negative self-referentiality and intense emotionality (e.g., somatic arousal) proposed in the emotion dysregulation model of Mennin and Fresco (2013,2014) in their emotion dysregulation model, decentering represents a metacognitive process that involves the capacity to observe items that arise in the mind (e.g., thoughts, feelings, memories, bodily sensations) with healthy psychological distance, greater self-awareness and perspective-taking (Safran and Segal, 1990; Fresco et al., 2007a,b; Bernstein et al., 2015). Depressed individuals tend to score lower on self-report (Fresco et al., 2007a) or objective behavioral measures (Shepherd et al., 2016) of decentering. Improvements in decentering consistently predict acute and enduring treatment effects for patients suffering from MDD (Fresco et al., 2007b), GAD (Hoge et al., 2015) and GAD (with and without MDD; Mennin et al., 2015, under review; Renna et al., under review) as well as the prevention of MDD relapse following prophylactic treatment with mindfulness based cognitive therapy (Bieling et al., 2012). However, few studies have examined the neurobehavioral underpinnings of decentering. Some studies, primarily with normative samples reveal patterns of neural activation in DN and SN networks consistent with decentering’s theorized role in reducing negative self-referentiality. For example, in a recent study examining the meta-awareness of mind wandering, low levels of meta-awareness were associated with greater activity in the medial and lateral anterior PFC, PCC and precuneus (Christoff et al., 2009). Similarly, in a sample of meditation practitioners, Hasenkamp et al. (2012) found that greater meta-awareness of mind wandering was linked to activity in the bilateral anterior insula and dorsal anterior cingulate cortex whereas mind wandering with low meta-awareness was associated with greater activation of the PCC, medial PFC, posterior parietal/temporal cortex, and parahippocampal gyrus (Hasenkamp et al., 2012).

In summary, GAD and ruminative MDD patients exhibit intense emotional experiences (e.g., somatic arousal) coupled with excessive negative self-referentiality and low ability to effectively decenter from their emotions and somatic responses (e.g., Mennin and Fresco, 2013), likely reflecting disruption in the DN and SN (e.g., insula). This potential endophenotype of patients who frequently evidence suboptimal treatment response to otherwise efficacious treatments motivated the development of Emotion Regulation Therapy (ERT), which represents a theoretically-derived, mechanism focused treatment designed to target and normalize these hypothesized neurobehavioral deficits (Fresco et al., 2013; Mennin et al., 2015, under review). Teaching skills that increase one’s capacity for decentering lies at the core of ERT and has been shown to mediate treatment gains (e.g., Mennin et al., under review).

Given the promising preliminary treatment efficacy evidence for ERT, the current study sought to demonstrate treatment-related neurobehavioral correlates commonly associated with mind-body phenomena that may also reflect this hypothesized endophenotype. The current study is a secondary analysis of the latest open label clinical trial of ERT (parent study) with findings reported elsewhere wherein 31 patients were treated with ERT (Renna et al., under review). Findings revealed impressive reductions in GAD (Hedges’ *g* = 4.05) and MDD (*g* = 2.82) severity following treatment. Similarly, patients evidenced reductions in disability (*g* = 1.40) as well as gains in perceived quality of life at post-acute treatment (*g* = 1.72). Finally, patients also evidenced clinical improvement on the ERT model variables relevant to the current study at post-acute treatment: worry (*g* = 2.79), anxious arousal (*g* = 1.82) and decentering (*g* = 2.60).

Thus, using ERT as a probe, we sought to examine the intrinsic functional connectivity (iFC) of the DN and the insula (as an index of the salience network) associated with treatment-related changes in theoretically motivated model variables (e.g., worry, anxious arousal, decentering). In particular, we used seed-based region of interest (ROI) analyses related to the DN (e.g., MPFC, PCC) and the insula (e.g., bilateral anterior and posterior insular cortices), using data acquired during resting state fMRI scans at pre-treatment. Based on previous findings and conceptualizations, we hypothesized that: (1) ERT will decrease negative self-referential processing and aversive body awareness (i.e., worry and hyper focus on bodily signals) and increase healthier self-consciousness and detached body awareness (i.e., decentering). We also hypothesized that; (2) changes in self-referencing (i.e., worry) and body awareness (i.e., anxious arousal) will be predicted by pre-treatment connectivity of specific networks (worry by DN areas and anxious arousal by insula); and (3) in contrast, more distributed network connectivity among nodes of the DN and the SN (i.e., insula) at pre-treatment will be associated with improvements in decentering.

## Method

### Participants

Participants consisted of 22 treatment seeking young adults drawn from a larger sample (*N* = 31; Renna et al., under review) of undergraduate and graduate students from a large, urban university, who completed baseline fMRI assessments and a 16-week trial of ERT (Mennin and Fresco, 2014). Participants were recruited through a variety of different strategies including direct referrals from an on-campus counseling center, fliers posted throughout campus, e-mail announcements sent to the entire student body, and through research staff handing out business cards to students on campus. Participants had a mean age of 21.9 years old (*SD* = 2.62, Range 18–29). Seventeen participants were female (77.3%). The sample was racially diverse: Caucasian (36.4%), African American (9.1%), Asian/Pacific Islander (22.7%), Other/mixed race (31.8%).

#### Inclusion/Exclusion Criteria

The main eligibility criterion was the presence of a primary or secondary GAD diagnosis (primacy was determined by clinical severity). In the current study, 16 patients had a primary diagnosis of GAD. Sixteen patients (72.7%) also met criteria for MDD; 14 (63.6%) patients met criteria for at least one additional anxiety disorder diagnosis. Other diagnoses included social anxiety disorder (*n* = 10), panic disorder (*n* = 6), specific phobia (*n* = 4), obsessive compulsive disorder (*n* = 3), post-traumatic stress disorder (*n* = 1). Participants were required to be stabilized on any psychotropic medications for a period of at least 3 months prior to the start of treatment (*n* = 1 receiving antidepressant medication) and could not be enrolled in any other form of psychological treatment during the acute phase of ERT (16 weeks). Finally, participants had to be free of active suicidal ideation/intent, psychosis, bipolar I disorder, primary anorexia or bulimia nervosa, somatoform disorders, or substance and alcohol dependance. Given the use of fMRI assessment, other exclusionary criteria included standard MRI contraindications (e.g., ferromagnetic implants; head trauma with loss of consciousness; tattoos above the elbow; pregnancy).

### Diagnostic Assessment

Current and lifetime psychiatric disorders were assessed with the *Structured Clinical Interview for DSM-IV* (SCID; First et al., 2002). Graduate students and senior research assistants, extensively trained on the diagnostic assessment protocol administered this assessment. A principal investigator and an independent assessor, both of whom were blind to the participant’s diagnoses assigned at the intake interview, then confirmed participants’ diagnoses. Reliability was high, with kappa ratings ranging from 0.708 to 1.000, demonstrating good to excellent reliability. Reliability for diagnoses of GAD was 100%, whereas MDD was 87.10%. Independent assessors, who remained blind to treatment status of patients, assessed clinical improvement at mid-treatment, post-acute treatment, as well as three-, and 9-months following the end of treatment.

### Treatment

ERT consists of 16-session individual weekly psychotherapy sessions completed within a 20-week span. The first half of the treatment (Phase I) focuses on psychoeducation and cultivating mindful emotion regulation skills. During these first eight sessions, participants are taught attention regulation (i.e., orienting, allowing) and meta-cognitive regulation (i.e., distancing/decentering, and reframing) skills. In particular, these skills include cue detection wherein individuals are instructed on how to better attend to emotional and motivational cues that arise in daily life so that these cues are noticed with greater acuity and closer to when they first arise. Cue detection is supported by training participants in a variety of meditation practices that improve attention and metacognitive capacities. These meditation practices are introduced to patients who then practice them each day. Briefer versions of these meditation practices are also introduced so that they can be utilized in both predicted and impromptu stressful situations to reduce a patient’s reliance on negative self-referentiality and behavioral responses associated with escape or avoidance. The second half of treatment (Phase II) focuses on context engagement, which involves developing a proactive approach towards life with the goal of living more consistently with one’s values through the use of imaginal exposures and internal dialog tasks. Here, therapists direct patients in conducting in-session exposure exercises where patients envision a situation, goal, or outcome that they desire but it presently missing from their lives. This imaginal exposure serves to elucidate the motivational inclinations for reward and approaching the goal as well as the motivations associated with protecting one’s self from the threat associated with taking the action and/or costs associated with not succeeding. By giving voice to these motivational inclinations, patients learn to decenter from the intensity of these pulls and derive a behavioral response that reflects a more optimal balance of risk and reward. More information regarding the structure and specific components of ERT are described elsewhere (see Fresco et al., 2013; Mennin and Fresco, 2014; Renna et al., under review).

Clinicians consisted of seven doctoral students in clinical psychology who were trained to administer ERT and received 2 h of weekly supervision. These modal number of cases treated by each clinician was 3 (*M* = 2.75; Range = 1–4). To establish adherence to the treatment protocol, all treatment sessions were audio recorded, and a team of research assistants, not involved in the administration of ERT or assessment of treatment effects, coded 40% of all cases, with 25% of these cases reviewed by a second coder to establish reliability. Reliability rates between the coders were 100%. Coders rated the accuracy of the frequency and skillfulness of actions taken by the study therapists. Overall, skillfulness ratings of the therapists coded were 98.4% (Range = 95%–100%), while frequency of actions consistent with the treatment protocol was 91.2% (Range = 71%–100%). The adherence ratings for this trial indicate that therapists uniformly delivered ERT with a high degree of adherence and fidelity. Examination of treatment effects associated with particular clinicians revealed equivalence for self-report and clinician-assessed clinical outcomes (*p*’s > 0.70) across the seven trial therapists.

### Clinical Outcomes

The *Penn State Worry Questionnaire* (PSWQ; Meyer et al., 1990) is a 16-item self-report measure of pathological worry with scores ranging from 16 to 80. This extensively used measure has excellent psychometric properties across numerous studies. Fresco et al. (2003) reported that scores greater or equal to 65 reliably identified patients with a diagnosis of GAD in a heterogenous sample of patients seeking outpatient treatment. Cronbach’s alpha in the current sample was good (*α* = 0.80).

The *Mood and Anxiety Symptom Questionnaire-Short Form (MASQ,* Watson and Clark, 1991*)* was designed to capture symptoms of anxiety and depression along dimensions of Watson and Clark (1991) tripartite model. Overall, four factors are derived—two of which demonstrate symptoms associated with anxiety and two with depression—and yield scores for four subscales: General Distress Anxiety, Anxious Arousal and, General Distress Depression, Anhedonic Depression. For each of these subscales, higher scores indicate greater anxious and depressive symptoms, respectively. Given the focus of the current study on somatic anxiety outcomes, only Anxious Arousal (MASQ-AA) was examined in the present study, which demonstrated excellent internal consistency (*α* = 0.91).

The *Experiences Questionnaire-Decentering Subscale* (Decentering; Fresco et al., 2007a) is an 11-item measure assessing the meta-cognitive strategy of decentering, or viewing oneself as separate from their emotional experience. Sample items include, “I can separate myself from my thoughts and feelings”, “I can observe unpleasant feelings without being drawn into them”, “I am consciously aware of a sense of my body as a whole”, and “I view things from a wider perspective”. The Decentering subscale has demonstrated strong psychometric properties and treatment sensitivity. Cronbach’s alpha in the current sample was good (*α* = 0.80).

### Procedure

The Institutional Review Board of the college approved all aspects of the study. Participants provided written informed consent for all procedures at the outset of study. At the initial intake visit participants were assessed for current and lifetime psychiatric history via the SCID interview and also completed a battery of self-report questionnaires delivered in paper-and-pencil format. Prior to the start of treatment, participants completed an independent assessment with a different interviewer who re-assessed the diagnoses that were of clinical threshold at the initial intake. Finally, participants completed the fMRI scan. Following the first eight sessions (i.e., mid-treatment) and after sixteen sessions (i.e., post-treatment), participants returned to the lab to complete another independent assessment and self-report questionnaire packet. They were also invited to complete another fMRI session post-treatment, but only the pre-treatment data are being analyzed for this study. Participants were compensated for all research related study visits.

### Analytic Plan

#### MRI Data Acquisition

Imaging data were collected on a 3.0T Siemens Allegra head-dedicated MRI scanner with a standard quadrature head coil at the NYU Center for Brain Imaging in New York, NY, USA. Scan sessions lasted 90 min during which participants completed a resting state fMRI scan (R-fMRI), and an anatomical scan, and three task-based scans (not examined in the current study). The resting state scan was always acquired prior to the task-based scans. During the 6-min resting-state sequence, participants were asked to keep their eyes open while a white crosshair was displayed on a black screen. The resting-state scan comprised 180 contiguous whole-brain functional volumes, acquired using a multi-echo echo planar imaging (EPI) sequence (repetition time = 2000 ms; echo time = 30 ms; flip angle = 90°; 33 slices; matrix = 64 × 64; voxel size = 3 × 3 × 4 mm). High-resolution T1-weighted MPRAGE structural images (TR = 2500 ms; TE = 3.93 ms, flip = 8°, 1 × 1 × 1 mm voxels) were acquired to facilitate localization and coregistration of functional data.

#### MRI Data Preprocessing

MRI preprocessing and data analysis were completed using an alpha version of an innovative software package: the Configurable Pipeline for the Analysis of Connectomes Version 3.9.1 (C-PAC^1^). CPAC is a configurable, open-source, Nipype-based^2^, automated processing pipeline for R-fMRI data. Preprocessing consisted of the following: slice time correction (first slice as reference, interleaved acquisitions, Fourier interpolation), 3D motion correction, despiking of extreme time series outliers using a continuous transformation function, spatial smoothing (FWHM = 6 mm), mean- based intensity normalization of all volumes by the same factor, and temporal band-pass filtering (0.01–0.1) in order to isolate the low-frequency BOLD fluctuations of interest. Structural images were registered to a common stereotaxic space (Montreal Neurological Institute, MNI) using Advanced Normalization Tools (Avants et al., 2011,^3^). Functional image registration was completed using Boundary Based Registration as implemented in FSL (Greve and Fischl, 2009). Single participant nuisance regression included linear and quadratic trends, 24 Friston motion parameters (Friston et al., 1996), and five CompCor signals (Behzadi et al., 2007). All analyses were Gaussian random field (GRF) corrected at *p* < 0.05, *Z* > 2.3.

As micromovements have been shown to potentially introduce artifactual correlations, mean framewise displacement (FD) was computed, per recently described methods (Power et al., 2012; Van Dijk et al., 2012). No subjects were excluded on the basis of motion (all had mean FD < 0.25 mm). To further control for the impact of motion on group results, each subject’s mean FD was included as a covariate in group-level analyses.

#### Region of Interest Selection

**Figure 1:**
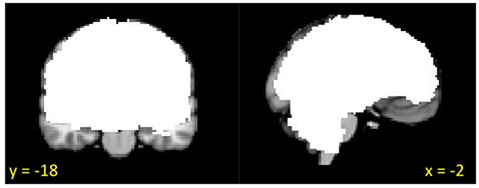
**Group mask (*n* = 22) indicating coverage of fMRI signal**.

**Figure 2:**
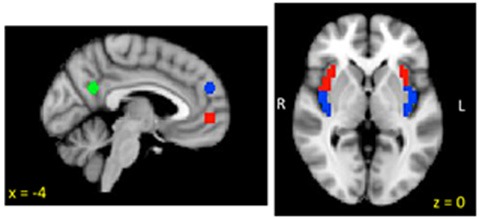
**Insula and default network (DN) regions of interest (ROIs)**.

To test study hypotheses, we utilized ROIs from the (DN) and the insula (see Figure 1). Three spherical ROI seeds (2 mm radius) were created to assess DN iFC based on Andrews-Hanna et al. (2010): the anterior MPFC (aMPFC) and PCC were selected because they represent hubs of the DN, and the dorsal (dMPFC) was selected to probe the dMPFC subsystem which has been implicated in social cognition, mentalizing and introspection about mental states (e.g., Wagner et al., 2016). Due to insufficient coverage of medial temporal and ventral prefrontal regions (see Figure 2), we were unable to examine the medial temporal lobe (MTL) subsystem. Anatomical bilateral posterior and anterior insula ROIs were taken from the two-cluster parcellation results from a recent multimodal examination of the functional organization of the insula (Kelly et al., 2012).

The mean time series of each ROI seed was calculated by averaging the time series of all voxels within each ROI. For each participant, ROI connectivity strength was assessed across the whole brain using Pearson correlations between the ROI time series and all other voxels in the brain. This resulted in individual Fisher’s *Z*-transformed participant-level maps of all voxels exhibiting significant iFC with the aMPFC, vMPFC and PCC, anterior insula (bilateral) and posterior insula (bilateral; GRF corrected: *p* < 0.05, *Z* > 2.3).

#### Group Level MRI Analyses

Three separate group-level analyses were conducted using a random-effects, ordinary least-squares model in FSL FEAT^4^ to assess the associations between iFC and worry, anxious arousal and decentering. Each model included three nuisance regressors (age, sex and mean FD), the pre-treatment measure of interest as a covariate, and post-treatment measure of interest as the primary predictor. Group-level analyses were conducted using cluster-level GRF theory for multiple comparison correction (*p* < 0.05, *Z* > 2.3) resulting in thresholded *Z*-score maps indicating clusters where iFC of each ROI was significantly related to the post-treatment variable of interest. To conduct additional *post hoc* analyses, we extracted the average partial regression coefficients for each significant cluster for each participant.

#### Corrections for Multiple Comparisons

Although all theoretically motivated and defensible, the analyses reported herein did use three ROI seeds associated with the DN and four ROI seeds associated with the salience network. Thus, to prevent the likelihood of Type I statistical errors, we report findings with *p* values less than 0.05 and make note of findings that do not survive a Bonferroni correction of 0.05/3 in the DN and 0.05/4 in the salience network.

## Results

### Pre-Treatment iFC Associated with Treatment-Related Changes in Worry

**Figure 3:**
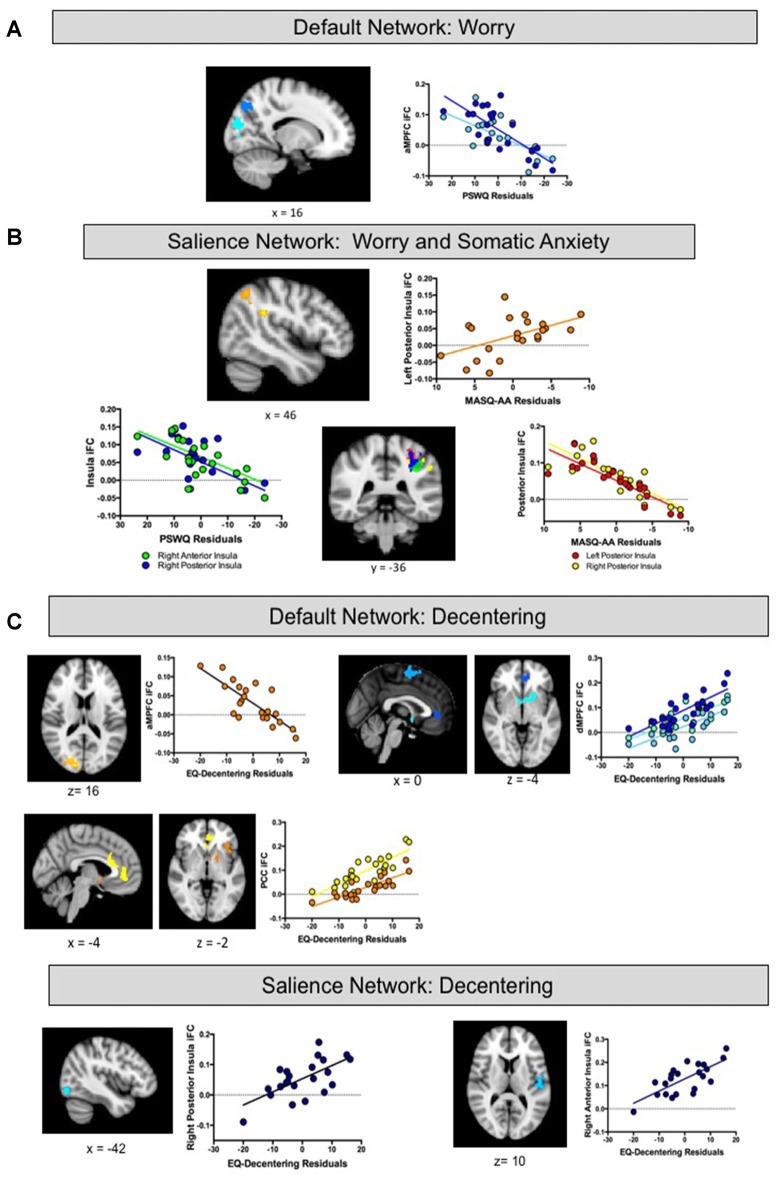
**(A,B)** Intrinsic functional connectivity (iFC) of default and salience regions associated with treatment-related changes in Worry and Somatic Anxiety Findings. **(C)** IFC of default and salience regions associated with treatment-related changes in Decentering. aMPFC, anterior medial prefrontal cortex region of interest; dMPFC, dorsal medial prefrontal cortex region of interest; PCC, posterior cingulate cortex region of interest.

Regression analyses estimated from changes in worry revealed associations with iFC of both the DN and insula. With respect to the DN, reduced iFC of the aMPFC with posterior regions including the precuneus and the occipital cortex, was associated with greater reductions (improvement) in worry following treatment (see Table 1 and Figure 3A). No significant results were observed for the PCC or dMPFC ROI analyses. With respect to the insula, greater reductions in worry following treatment were associated with weaker iFC of right anterior and posterior insula with the superior parietal lobe (see Figure 3B). A similar cluster was observed for the right anterior insula but this did not survive Bonferroni correction (*p* > 0.0125). No significant clusters emerged for the left insula.

**Table 1:** **Clusters with significant associations in relation to clinical change in worry, somatic anxiety and decentering**.

	Cluster size	*x*	*y*	*z*	Max *Z*	*p*
**Worry with DN ROIs**
*aMPFC*
Precuneus	368	18	−78	42	4.59	0.00269*
Occipital cortex	539	−8	−80	18	4.31	0.000119*
**Worry with insula ROIs**
*Right posterior insula*
Superior parietal cortex	241	−30	−38	40	3.77	0.00897*
*Right anterior insula*
Inferior parietal cortex	189	−50	−28	42	4.3	0.0484
**Somatic anxiety with insula ROIs**
*Right posterior insula*
Superior parietal lobe	251	−34	−36	40	3.85	0.00517*
*Left posterior insula*
Superior parietal lobe	433	−32	−38	48	3.9	0.000125*
Inferior parietal lobe	269	46	−38	28	2.24	0.00573*
**Decentering with DN ROIs**
*aMPFC*
Occipital pole	336	16	−90	16	3.68	0.00407*
*dMPFC*
Rostral ACC	225	0	42	0	4.62	0.0486
Superior frontal gyrus	226	−2	−2	68	4.18	0.0475
Left anterior insula/inferior frontal gyrus	363	−26	12	−16	3.81	0.00258*
*PCC*
Striatum (caudate and putamen)	537	−20	8	−8	3.48	0.000329*
Rostral ACC	606	2	36	4	3.95	0.000113*
**Decentering with insula ROIs**
*Right posterior insula*
Lateral occipital cortex	245	−42	−80	−14	3.77	0.021
*Right anterior insula*
Central opercular cortex	220	−42	−22	18	4.31	0.0361

Note: *Denotes findings that survive a Bonferroni correction for multiple comparisons.

### Pre-Treatment iFC Associated with Treatment-Related Changes in Somatic Anxiety

Regression analyses estimated from changes in somatic anxiety revealed no significant findings for any of the DN ROIs. However, similar to the worry analyses, reduced iFC between bilateral posterior insula ROIs and a cluster in the superior parietal lobe was associated with greater reductions in somatic anxiety. See Table 1 and Figure 3B. Additionally, greater iFC between the left posterior insula and inferior parietal lobe was associated with lower post-treatment anxious arousal, as shown in Figure 3A. No results were found for the right or left anterior insula.

### Pre-Treatment iFC Associated with Treatment-Related Changes in Decentering

Finally, regression analyses estimated from changes in decentering revealed associations with iFC of both the DN and insula. Reduced iFC between the aMPFC and a cluster in occipital lobe was associated with higher post-treatment decentering scores (greater improvement). Of note, this cluster overlapped with that obtained in the aMPFC iFC analysis for worry. Multiple regions emerged from the dMPFC iFC analysis. Here, greater iFC between dMPFC and the left anterior insula/inferior frontal gyrus was associated with higher post-treatment decentering. Clusters were also detected in the rostral ACC extending to striatum and superior frontal gyrus but these did not survive correction for multiple comparisons (*p*’s > 0.017). Finally, greater connectivity between the PCC ROI and a cluster extending from dorsal to rostral ACC and a cluster encompassing striatum and anterior insula was associated with greater improvements in decentering. With respect to the insula, greater iFC between the right posterior insula and lateral occipital cortex as well as iFC between the right anterior insula and central opercular cortex were associated with higher decentering at post-treatment. However, these findings were no longer significant after controlling for multiple comparisons (*p*’s > 0.0125). No significant results were found for the left insula. See Table 1 and Figure 3C.

## Discussion

The present study took a novel approach to examining the neural predictors of specific outcomes of ERT for GAD patients with and without comorbid MDD. In particular, much of the psychological theorizing about the nature of GAD and MDD in terms of disruption of mind and body has been predicated on a functional relationship between the kinds and intensity of visceral and psychological sensations that one experiences (i.e., motivational intensity) and the ways by which one attempts to respond to their arising (i.e., negative self-referentiality; Borkovec et al., 2004; Nolen-Hoeksema et al., 2008; Watkins, 2008; Newman and Llera, 2011; Mennin and Fresco, 2013, 2014; Olatunji et al., 2013). Extending this model to an examination of neurobehavioral systems that subserve emotion and motivation (i.e., salience network) as well as self-referential mentation (i.e., DN) motivated the current study, in which we focused on two constructs characteristic of the pathology of GAD and MDD, worry and somatic anxiety, and one treatment-related construct associated with reduced negative self-referentiality, decentering. From a neural perspective, we hypothesized that change in these variables would be differentially associated with the iFC of insula-based and DN regions. Specifically, because worry is regarded as a destructive form of self-referentiality, we hypothesized that changes in worry would be related primarily to DN connectivity. Conversely, we hypothesized that changes in somatic anxiety would be associated primarily with pre-treatment insula iFC. Finally, given that decentering is a metacognitive capacity associated with reductions in negative self-referentiality and a consistent mechanism of clinical improvement, we predicted that gains in decentering would be associated with both DN and insula iFC at pre-treatment.

Overall, we found some distinctions between iFC patterns associated with improvements in worry and somatic anxiety, although there was greater overlap than predicted. As hypothesized, DN iFC was associated with treatment-related reductions in worry but not in somatic anxiety. Specifically, individuals with weaker iFC between the anterior MPFC and posterior regions demonstrated greater improvements in worry. Conversely, insula iFC was not unique to changes in somatic anxiety. Although individuals with weaker pre-treatment iFC between bilateral posterior insula and superior parietal lobe showed greater improvements in somatic anxiety, iFC of right anterior and posterior insula with same regions was also associated with changes in worry. This pattern of results bears some resemblance to the findings of Kaiser et al. (2015), who in their recent meta-analysis of MDD, reported abnormal connectivity patterns in both the dorsal and ventral attention networks, potentially reflecting altered or biased salience monitoring. Similarly, Andreescu et al. (2014) found that broad scale hyperconnectivity of the insulae was associated with greater GAD severity. Further, Paulus and Stein (2010) postulate a role of negative self-referentiality in aberrant insula activity. The findings from the current study are largely consistent with this interpretation and suggest that weaker pre-treatment iFC of the insula (both posterior and anterior ROIs) with regions of the dorsal attention network (i.e., post-central gyrus, superior parietal lobe) is a non-specific indicator of ERT treatment gains.

Psychological models have identified decentering as a metacognitive process that occurs naturally in individuals but can be cultivated by psychosocial interventions, especially interventions that include training in mindfulness meditation (e.g., Bernstein et al., 2015). Decentering is central to the ERT treatment model as it represents an important mechanism of change (Mennin and Fresco, 2013, 2014; Mennin et al., under review). Bernstein et al. (2015) posit that decentering helps individuals reduce time spent engaging in negative self-referentiality and allowing them to better attend to cues associated with goal directed behavior; thus, we postulated that ROIs from both the default and salience networks would be associated with ERT linked gains in decentering. Consistent with our initial predictions, clinical gains in decentering were associated with pretreatment iFC of several ROIs from the DN and the insula. With respect to the DN, findings revealed that reduced connectivity between the anterior MPFC and the occipital pole at baseline was associated with greater increases in decentering over the course of ERT. This finding is comparable to that of reduced anterior MPFC—occipital cortex iFC predicting improvement in worry suggesting overlap in these constructs (or in treatment gains related to these constructs). As reflected in the operational definition of decentering and the content of items in the decentering subscale, decentering is believed to be a metacognitive capacity that leads to less self-referentially biased awareness of exteroceptive and interoceptive cues. Similarly, previous task-based work has shown that reduced co-activation of these regions predicts greater prevention of relapse in patients with remitted depression (Farb et al., 2011). It may be that reduced connectivity between the aMPFC and visual processing regions is associated with less negative self-referentiality and greater engagement of imagery (i.e., potentially indicating emotional processing; Foa and Kozak, 1986). Individuals with GAD are suggested to engage in verbally-based processing (i.e., worry) as a strategy for avoiding intense and aversive internal experiences (Borkovec et al., 2004). Similarly, individuals with MDD show reduced concreteness during rumination (Watkins and Moulds, 2007), and individuals with MDD and anxiety disorders, including GAD, show a reduced ability to generate prospective positive imagery (Morina et al., 2011). Greater imagery engagement may indicate emotional processing (e.g., Foa and Kozak, 1986) and therefore be important in treating disorders involving a high level of perseverative cognitive processing—for example, an interpretation modification bias paradigm found that imagining positive future events, vs. thinking about their meaning in words, had a protective effect against a subsequent negative mood induction (Holmes et al., 2009).

Greater pretreatment connectivity of the dMFPC and PCC ROIs with various regions were associated with greater gains in decentering. One area that evidenced greater connectivity to the PCC was the rostral ACC. Although the ACC is broadly associated with many neural functions including conflict monitoring and adaptation (i.e., confronting and resolving situations marked with conflicting information, Botvinick et al., 2001; Etkin et al., 2006), particular connectivity between the dMPFC and the rostral ACC has been implicated in the conscious appraisal of emotion (Etkin et al., 2011), which seems consistent with one’s ability to decenter from negative emotions. Likewise, connectivity between the rostral ACC and the MPFC may simply reflect its role within the default network (e.g., Buckner et al., 2008). Interestingly, recent findings implicate hypoactivation of the rostral ACC especially in conjunction with the MPFC in emotion regulation deficits in GAD (Klumpp et al., 2011; Mochcovitch et al., 2014). Recent theories suggest that the rostral ACC may be a node for more implicit emotion regulation, especially when not co-activated with aspects of the dorsal attention network (i.e., DLPFC, dorsal ACC; Christoff et al., 2011). Thus, this greater connectivity observed between the rostral ACC to the PCC may reflect increased ability to notice and resolve emotionally laden self-relevant information, which in turn could facilitate treatment-linked gains in decentering.

With respect to the observed connectivity between the dMPFC with the left anterior insula/IFG cluster, these areas have been implicated in effective emotion regulation especially in the context of affect labeling (e.g., putting feeling into words; Lieberman et al., 2007) among individuals with high trait mindfulness (Creswell et al., 2007). Such connectivity may suggest that adaptive emotion regulation requires coordination between the default and salience networks. Although no studies have formally examined decentering in the context of affect labeling, these two constructs are conceptually similar. Thus, these patterns of findings may indicate that pretreatment iFC in these regions facilitates gains in decentering for patients receiving ERT.

Findings from the current study add to a growing body of research demonstrating the clinical efficacy and hypothesized mechanism model of ERT (Mennin and Fresco, 2013, 2014). For instance, in the context of a randomized clinical trial using a modified attentional control as the comparator, Mennin et al. (under review) reported acute and enduring treatment effects for patients receiving ERT for a broad range of clinical outcomes associated with GAD and MDD (Hedge’s *g*’s of 0.48–1.50). Treatment effects in that trial were mediated by gains in self-reported decentering (e.g., Fresco et al., 2007b). Similarly, one mechanism finding from an earlier ERT trial consistent with the present findings relates to heart rate variability, an index of parasympathetic flexibility (Porges, 2001; Thayer et al., 2012). Heart rate variability was assessed while watching a fearful film in a subset of ERT patients from prior trials. At pre-treatment, patients displayed a flattened response throughout the experimental period, suggesting reduced cardiac flexibility. At mid-treatment, patients displayed a quadratic pattern of vagal withdrawal (i.e., reactivity) and vagal rebound (*d* = 0.81). This relative normalization of parasympathetic flexibility from pre- to mid-treatment predicted treatment gains in diagnostic severity, anxiety and mood symptoms (Mennin et al., in preparation).

Although these findings are encouraging, they must be regarded as preliminary given some notable limitations. First, our sample was relatively small (*N* = 22), which prevented us from examining specific effects in patients with GAD with and without MDD to assess the impact of this comorbidity. It also prevented more sophisticated hierarchical analyses that would include all three constructs in one model. Our small sample, comprised mostly of women, also did not allow us to adequately test gender differences in treatment response and neural patterns. However, this imbalance in gender distribution favoring women is consistent with treatment seeking patterns for all anxiety disorders including GAD (e.g., McLean et al., 2011). Second, the findings are based upon patients receiving ERT in an open-label format. Thus, this trial lacked a comparator treatment to contrast the potential association of pre-treatment neural activations to treatment changes uniquely attributable to ERT. These limitations speak to the need of a larger ERT trial with a proper comparator arm and an assessment schedule with multiple fMRI visits—thereby allowing for a careful examination of ERT specific neural changes associated with clinical improvement for patients suffering from GAD and MDD. Finally, our MRI acquisition did not allow us to adequately image and examine the MTL subsystem including the bilateral amygdala, which has been implicated in GAD and MDD (Roy et al., 2013; Oathes et al., 2015). Although the specific hypotheses of the current study were not unduly affected by this limitation, future studies will benefit from MRI acquisition that can simultaneously examine all the neural systems associated with GAD and MDD.

Despite these limitations, the present findings add to the growing body of research implicating disruptions in the DN and salience networks in GAD and MDD and demonstrate the association of functional connectivity in these networks to patterns of treatment-related changes in central components of these disorders (i.e., worry, somatic anxiety) and their amelioration (i.e., decentering).

## Author Contributions

DMF, AKR, SS, EG-L and DSM: substantial contributions to the conception or design of the work; DMF, AKR, SA, SS, EG-L, CL and DSM: the acquisition, analysis, or interpretation of data for the work, drafting the work or revising it critically for important intellectual content, final approval of the version to be published, agreement to be accountable for all aspects of the work in ensuring that questions related to the accuracy or integrity of any part of the work are appropriately investigated and resolved.

## Funding

CUNY Collaborative Incentive Research Grant (CIRG), Grant #2054. PSC-CUNY Enhanced Research Award, Grant #65797-0043; National Institutes of Health (NIH) MBRS-RISE Program at Hunter College, Grant #GM060665; Doctoral Student Research Grant, City University of New York, Graduate Center; NIH 1R01HL119977-01 & 1P30NR015326-01.

## Conflict of Interest Statement

The authors declare that the research was conducted in the absence of any commercial or financial relationships that could be construed as a potential conflict of interest.

## Footnotes

^1^http://fcp-indi.github.io/

^2^http://nipy.org/nipype/

^3^http://www.picsl.upenn.edu/ANTS

^4^https://fsl.fmrib.ox.ac.uk
